# Analysis of the expression profile of Dickkopf-1 gene in human glioma and the association with tumor malignancy

**DOI:** 10.1186/1756-9966-29-138

**Published:** 2010-10-28

**Authors:** Youxin Zhou, Fang Liu, Qinian Xu, Xiuyun Wang

**Affiliations:** 1 Department of neurosurgery, The First Affiliated Hospital of Soochow University, Suzhou 215006, Jiangsu, China; 2 Department of neurosurgery, Changzhou NO.2 People's Hospital, Changzhou 213003, Jiangsu, China

## Abstract

**Background:**

Gliomas represent the most common primary malignant brain tumors, yet little is known about the molecular pathogenesis of these tumors. The highly-regulated Wnt signal transduction pathway is essential for normal developmental processes, and defects in the pathway are closely linked to oncogenesis. Dickkopf-1 (DKK-1) is a secreted protein that acts as a potent inhibitor of the Wnt pathway. The aim of this study was to examine the expression profile of DKK-1 gene in human glioma and its association with tumor malignancy.

**Methods:**

We determined the expression levels of DKK-1 transcript and protein in 12 glioblastoma cell lines, medulloblastoma cells, low-grade glioma cells, and human astrocyte cells by semiquantitative RT-PCR and ELISA. A total of 47 tumor biopsy specimens and 11 normal brain tissue samples from patients with cerebral trauma internal decompression were embedded in paraffin blocks and used for immunostaining. Twenty-six primary tumors and 7 corresponding brain samples were stored in liquid nitrogen and used for RT-PCR. We further examined serologic concentrations and cerebral fluid levels of DKK-1 in patients with tumors.

**Results:**

DKK-1 could only be detected in 12 human glioblastoma cell lines, not in a panel of other tumor and normal cell lines. The difference between glioma patients and healthy individuals was significant. Kendall's tau-c association analysis also revealed the increased DKK-1 protein expression in tumor tissues of higher pathologic classification. The levels of cerebral fluid DKK-1 protein were significantly higher in glioma patients than in healthy donors or in neuronal benign tumor patients, suggesting that the DKK-1 molecule in cerebral fluids can be applicable to detect the presence of glioma and be developed as a novel prognostic treatment.

**Conclusion:**

The Wnt antagonist DKK-1 gene may have important roles in glioma tumorigenesis and act as a novel biomarker in human malignant glioblastoma.

## Background

The Wnt signaling cascade plays a critical role in cell patterning, proliferation, and fate-determination during embryogenesis, In humans, the Wnt glycoproteins comprise a family of extracellular ligands that regulate homeostasis and development by binding to Frizzled (Fz) receptors and the LDL receptor-related protein 5/6 (LRP5/6) located at the plasma membrane [1]. Wnt glycoproteins signal through canonical and noncanonical pathways. The canonical Wnt pathway involves the stabilization and accumulation of β-catenin in the cytoplasm, its subsequent nuclear translocation and gene regulation. Accumulation of β-catenin in the cytosol is caused through inhibition of its proteasome-targeting phosphorylation by glycogen synthase kinase-3, which forms a complex with the tumor suppressor *adenomatous polyposis coli *(APC) and Axin proteins. And in the nucleus, β-catenin associates with T-cell factor/lymphocyte enhancer factor (TCF/LEF) family of transcription factors to stimulate the expression of multiple Wnt target genes including c-myc, c-jun, and cyclin D1 [2,3]. Defects in this highly regulated signal transduction pathway have been closely linked to oncogenesis, i.e. early activation by mutation in APC or β-catenin occurs in a proportion of carcinomas [2,4]. It is also thought that an important component of cancer induction and progression may be the loss of control over β-catenin levels [5]. Unlike the canonical Wnt pathway, non-canonical pathways transduce signals independent of β-catenin and include the Wnt/Ca^2+ ^pathway, the planar cell polarity (PCP) pathway in *Drosophila*, the convergent extension pathway in vertebrates, and the JNK pathway, a potential mediator of noncanonical signaling with unclear roles [6]. Noncanonical pathways lead to the activation of the small GTPases Rho and Rac, or kinases such as JNK and PKC, or to modulation of Ca^2+ ^levels [4,7].

Wnt signals are extracellularly regulated by several natural antagonists that can be classified into two broad groups of molecules, both of which prevent Wnt-Fz interaction at the cell surface [8]. The first group consists of proteins that bind directly to the Wnt ligand and include Wnt inhibitory factor (WIF-1), the secreted frizzled-related protein (sFRP) family, and Cerberus. The second group includes members of the DKK family, secreted glycoproteins which inhibit the Wnt pathway in a manner distinct from the other Wnt antagonists and do not prevent Wnt from associating with Fz receptors [8,9]. Previous results have demonstrated that Wnt must bind to both LRP5/6 and Fz in order to form a functional ligand-receptor complex that activates the canonical Wnt/β-catenin pathway [9]. DKK-1, the most well-characterized member of the latter group of antagonists, blocks Wnt signaling by binding to LRP5/6 co-receptor and the membrane-anchored molecule, Kremen, thereby inducing LRP endocytosis that inhibits the Wnt-induced Fz-LRP5/6 complex formation and triggers the consequent removal of the DKK-1-LRP5/6 complex from the plasma membrane during mid blastula transition [2,10-13].

It is thought that the antagonistic effect of DKK-1 is specific for the canonical Wnt/β-catenin signaling pathway [11,14]. However, one recent report has demonstrated that restoration of DKK-1 expression suppresses cell growth and induces apoptotic cell death in β-catenin-deficient mesothelioma cell lines H28 and MS-1. Moreover, a small-molecule inhibitor of JNK inhibited the apoptosis induced by DKK-1 overexpression in these cells. Similarly, DKK-1 sensitized HeLa cervical carcinoma cells to apoptosis, acting as a suppressor of cell transformation. This effect of DKK-1 was not due to inhibition of β-catenin/TCF4-regulated transcription, as the cellular localization of β-catenin and activities of targets in the Wnt/β-catenin pathway remained unchanged [15]. These data suggest that DKK-1 may be able to antagonize Wnt signaling and have additional tumor suppressive effects through β-catenin-independent non-canonical pathways (i.e., the Wnt/JNK pathway).

Glioma is one of the most lethal malignancies of the human brain and is the leading cause of cancer-related death in the world. Despite some advances in early detection, most of the patients are at advanced stages at the time of diagnosis, and the prognosis of them still remains poor. In spite of the use of modern surgical techniques combined with various treatment modalities, such as radiotherapy and chemotherapy, the overall 5-year survival rate of glioma still remains at ~20%. Although several tumor markers are elevated in serum of glioma patients, no tumor marker has been sufficiently useful for detection of glioma at potentially curative stage, and a limited number of practical prognostic biomarker are presently available for selection of treatment modalities for individual patients.

Nowadays, the interaction of genes and environment is widely investigated by a combination of the molecular biology, cell biology, and genetic approach. It has been demonstrated that the progression and development of glioma is closely-related with the overexpression of several oncogenes and inactivation of tumor suppressor genes, however, the specific molecular mechanism remains largely unknown. Thus, the identification of putative genes and characterization of the relationship between changes of gene functions and progression of glioma in different stages are urgently need for isolating potential molecular targets for diagnosis, treatment, and/or prevention of glioma. In the current study, we analyzed the expression of DKK-1, an antagonist of Wnt signaling, in clinical glioma materials and cell lines at the mRNA and protein level. We also detected its expression in serum and cerebrospinal fluid of glioma patients.

## Materials and methods

### Cell lines, patients, and tumors

The 14 cancer cell lines used in this study included twelve glioblastomas (U251, SF767, SF295, T98G, MGR1, MGR2, MGR3, SKMG-1, SKMG-4, UWR7, UW-28, and SKI-N2), one medulloblastoma (D341), and one low-grade glioma (SHG-44). All cells were grown in monolayer in appropriate medium supplemented with 15% fetal calf serum (Hyclone, Logan, UT) and cultured at 37°C in a humid incubator with 5% CO_2_. Human astrocyte cells were used as a normal control.

A total of 47 paraffin-embedded primary tumors and 11 normal brain tissue (internal decompression in cerebral trauma) samples and used for semiquantitative reverse transcription-PCR and immunostaining had been obtained from 58 patients (30 female and 28 male patients; median age of 45.5 with a range of 11 to 74 years) undergoing curative surgery at the First Affiliated Hospital of Soochow University (Suzhou, China). A total of 26 tumor biopsy specimens and 7 corresponding normal brain tissue samples stored in liquid nitrogen (14 female and 19 male patients; median age of 47.4 with a range of 13 to 79 years) had also been obtained earlier from patients undergoing curative surgery at the First Affiliated Hospital of Soochow University (Suzhou, China) with informed consent. Clinical stage was judged according to the 2007 WHO classification of tumors of the central nervous system [16]. The use of all clinical materials in this study was approved by individual institutional Ethical Committees.

### Serum and cerebrospinal fluid samples

Serum samples were obtained with written informed consent from 8 healthy individuals and from 12 spongioblastomas, 6 low-grade gliomas, and 20 benign tumor patients in their neuronal system, i.e. the pituitary tumor, meningioma, nerve sheath tumor, and acoustic nerve tumor. The median age of these samples (20 males and 26 females) was 50.1 with a range of 26 to 79 years. Cerebral fluid samples from a total of 36 cancer patients and 6 healthy control individuals were also selected with informed consent from 26 males and 16 females (median age of 48.9 with a range of 26 to 79 years). These 36 cancer cases included 14 spongioblastomas, 11 low-grade gliomas, and 11 patients with benign tumor in the neuronal system (pituitary tumor, meningioma, nerve sheath tumor, acoustic nerve tumor, etc.). The serum and cerebrospinal fluid samples in this study were obtained at the time of diagnosis, centrifuged, and the supernatants were stored in liquid nitrogen.

### RNA preparation and cDNA synthesis

Total cellular RNAs from cell lines and tissues were extracted and purified by using the Trizol reagent (Invitrogen, Inc.) according to the protocol of the supplier. Before RNA extraction, individual tissue samples were preexamined by frozen section histologic examination to document the histopathologic appearance of the specimen. About 10 μg total RNA from each sample was reversely transcribed to single-stranded cDNAs using random hexamers (Shanghai Sangon, Inc.) as primer and M-MLV reverse transcriptase (Promega, Inc.).

### Semiquantitative RT-PCR analysis of hDKK-1 expression

Analysis of the hDKK-1 mRNA expression was performed by a semiquantitative RT-PCR assay on Perkin Elmer PCR system 9600 using synthesized hDKK-1-specific primers together with primers for the house-keeping gene GAPDH as an internal control to ensure RNA quality and loading accuracy. Primer sequences were as follows: DKK-1, 5'-TCACGCTATGTGCTGCCCCG-3' and 5'-TGAGGCACAGTCTGATGACCGGA-3', product size 223 bp; and GAPDH, 5'-AGAAGGCTGGGGCTCATTTG-3' and 5'-AGGGGCCATCCACAGTCTTC-3', product size 258 bp. PCRs were optimized for the number of cycles to ensure product intensity to be within the linear phase of amplification. The PCR protocol consisted of an initial denaturation step of 95°C for 7 minutes, followed by 32 cycles of a three-step program of 94°C for 30 seconds, 56°C for 30 seconds, and 72°C for 45 seconds, and a final extension step of 72°C for 7 minutes. The PCR was performed in a final volume of 25 μl in the presence of 2.0 mM MgCl_2_, 0.75 U of *Taq *polymerase in PCR buffer, and 5 pmol of the hDKK-1 and GAPDH primers. PCR products were separated and analyzed on 1.5% agarose gels.

### Elisa

Levels of DKK-1 in cell medium, cell lysate, serum, and cerebral fluid were measured by ELISA with a commercially available enzyme test kit (R&D Systems, Inc.) according to the supplier's recommendations. First, a rabbit polyclonal antibody specific to DKK-1 was added to a 96-well microplate as a capture antibody and incubated overnight at room temperature. After washing away any unbound antibody, 0.75% BSA was added to the wells and incubated for at least 1 h at room temperature for blocking. After a wash, 3-fold diluted sera were added to the wells and incubated for 2 h at room temperature. After washing away any unbound substances, a biotinylated polyclonal antibody specific for DKK-1 was added to the wells as a detection antibody and incubated for 2 h at room temperature. After a wash to remove any unbound antibody-enzyme reagent, horseradish peroxidase (HRP)-streptavidin was added to the wells and incubated for 20 min. After a wash, a substrate solution was added to the wells and allowed to react for 20 min. The reaction was stopped by adding 50 μL of 2 N sulfuric acid. Color intensity was determined by a photometer at a wavelength of 490 nm, with a reference wavelength of 570 nm. Differences in the levels of DKK-1 between different groups were analyzed by t test. Significance was defined as P < 0.05.

### Immunohistochemistry

To investigate the DKK-1 protein in clinical samples that had been embedded in paraffin blocks, we stained the sections as previously described [17]. Briefly, 3.3 μg/mL of a rabbit polyclonal anti-hDKK-1 antibody (Santa Cruz Biotechnology) were added to each slide after blocking of endogenous peroxidase and proteins, and the sections were incubated with biotin-labeled anti-rabbit IgG as the secondary antibody. Substrate-diaminobezidine (DAB) was added, and the specimens were counterstained with hematoxylin.

### Statistical analysis

Statistical analyses were done using the SAS6.12 statistical program. Kendall's tau-c association analysis was applied between DKK-1 expression and pathologic tumor classification. We also analyzed positive DKK-1 expression rate in glioblastoma and normal brain tissues by chi-square test. Significance was defined as P < 0.05.

## Results

### Differential expression of DKK-1 mRNA and protein in various cell lines

**Figure 1:**
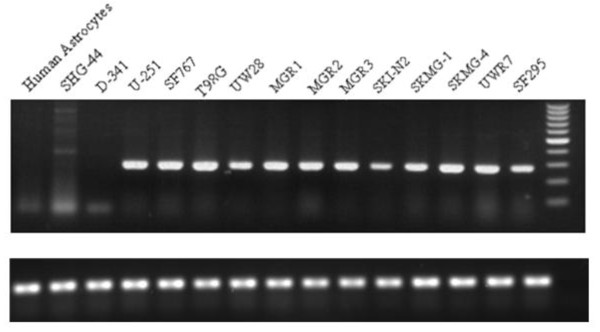
Expression of DKK-1 mRNA  in glioblastoma cell lines was higher than that in control by using semi-quantitative RT-PCR.

We first sought to identify the differential expression of the DKK-1 gene in 12 glioblastoma cell lines, medulloblastoma cells, low-grade glioma cells, and human astrocytes as a control using semi-quantitative RT-PCR analysis (Figure 1). In glioblastoma cell lines UW-28, SKI-N2, and SF295, DKK-1 mRNA expression was relatively lower as compared with other glioblastoma cells. Concentration of DKK-1 protein was also determined by ELISA in culture medium and cell lysate of these 14 cell lines (Table 1). U251 cells have the highest levels of DKK-1 expression in both of the culture medium and cell lysate, while glioblastoma cell lines SKMG-4 and UW-28 have the lowest DKK-1 levels in the culture medium and cell lysate, respectively. Following normalization and statistical analysis of fluorescence intensity data by t test, we identified that the difference of DKK-1 protein expression was significant between the culture medium and cell lysate in 12 glioblastoma cell lines (p < 0.05), consistent with the fact that DKK-1 was a secreted peptide shown previously to influence cell growth, differentiation and apoptosis by inhibiting Wnt signaling [18]. It should also be noted that the very low expression level of DKK-1 mRNA was not in concordance with the higher level of its protein in SKI-N2 cells. Expression of DKK-1 mRNA and protein was undetectable in medulloblastoma cells, low-grade glioma cells, and human astrocytes. Thus, DKK-1 can serve as a marker for diagnosis of glioma through detecting the expression of the protein and mRNA of DKK-1.

**Table 1:** Levels of DKK-1 expression were detected in the culture medium and cell lysate of all 14 cancer cell lines by ELISA

Cancer cell lines and control	Concentration of DKK1 (pg/ml)	
***Normal cell**s*		
Human astrocytes	0	0
***Low-grade glioma cell line***		
SHG-44	0	0
***Medulloblastoma cell line***		
D341	0	0

***Glioblastoma cell lines***	Culture medium*	Cell lysate**
U251	18238	4917
SF767	5760	729
T98G	1558	258
UW-28	2390	45
MGR1	1089	151
MGR2	3826	434
MGR3	3901	375
SKI-N2	766	260
SKMG-1	6691	2192
SKMG-4	301	72
UWR7	5290	910
SF295	8628	1780

* and ** indicate the respective concentration of DKK-1 protein tested in the culture medium and cell lysate.

### DKK-1 expression in tumors and normal tissues

**Figure 2:**
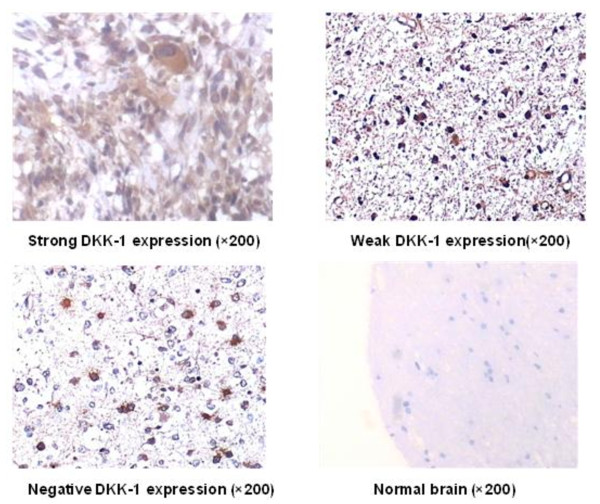
**Different hDKK-1 expression levels in tumor and healthy brain tissues analyzed by immunohistochemistry**.

**Figure 3:**
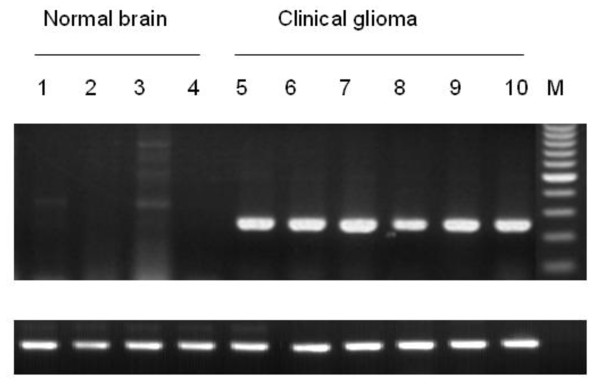
**Expression of DKK-1 was detected in selected tissue samples by RT-PCR**.

To identify the association of DKK-1 expression with pathologic tumor classification, we did DKK-1 expression profile analysis in patients at various clinical stages of glioma and in healthy controls. We screened 58 tissue samples embedded in paraffin blocks by immunohistochemistry and identified expression of DKK-1 protein located at cytoplasm (with granular appearance) of the great majority of the glioma samples examined (Table 2). The proportion of the DKK-1-positive cases was 91.5% for glioma (43 of 47). Representative data are shown in Figure 2. The difference between glioma patients and healthy individuals was significant (p < 0.05). Kendall's tau-c association analysis also revealed the increased DKK-1 protein expression in tumor tissues of higher pathologic classification (r_τ _= 0.3178, P < 0.01) (Table 3). The relatively high false-positive rate here (2 of 11) may be eliminated by testing more normal volunteers or measuring more tumor markers to improve overall sensitivity for detection of glioblastoma. We subsequently confirmed by means of semiquantitative RT-PCR experiment overexpression of DKK-1 mRNA in 26 tumor tissues frozen in liquid nitrogen, but its transcript was hardly detectable in any other normal tissues (P < 0.05) (Figure 3). These observations demonstrated that DKK-1 was a novel molecule that can be applicable to detect presence of glioma at an early stage and thus help us develop novel treatments based on the biological characteristics of tumor cells.

**Table 2:** DKK1-1 expression in glioma and corresponding normal brain tissues

	DKK-1expression			
	Strong (++)	Weak (+)	Negative (-)	Total
Glioblastoma tissue	28	15	4	47
Normal brain tissue	0	2	9	11

**Table 3:** Correlation between DKK-1 expression in different tumor stages and pathologic tumor classification

Stage	DKK-1expression			
	
	Strong (++)	Weak (+)	Negative (-)	Total
I	1	2	2	5
II	10	9	1	20
III	13	3	1	17
IV	4	1	0	5

### Serologic concentrations and cerebral fluid levels of DKK-1 in patients with tumors

**Figure 4:**
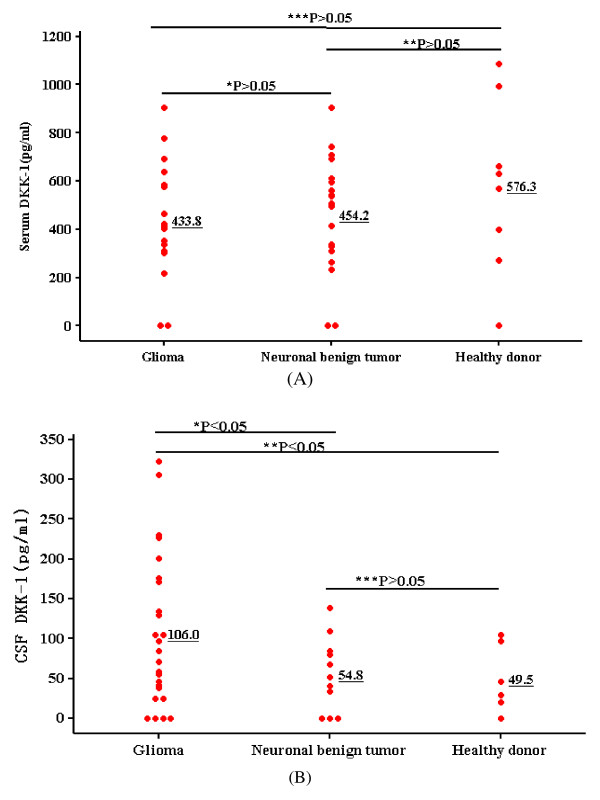
**DKK-1 concentration in sera (A) and cerebral fluid (B) samples determined by ELISA in patients with tumors and in healthy controls**. *, difference between the glioma group and neuronal benign tumor group. **, difference between the glioma group and normal control group. ***, difference between the neuronal benign tumor group and healthy control group. The DKK-1 concentration in cerebral fluid is increased in glioma, and differences may exist among different glioma grades, suggesting the role of DKK-1 in glioma pathogenesis.

Because DKK-1 encodes a secreted protein, we investigated the DKK-1 protein secreted into sera of patients with glioma or neuronal benign tumor and healthy individuals. ELISA experiments detected DKK-1 protein in serologic samples from 18 patients with spongbioblastoma or low-grade glioma, 20 benign tumor patients in their neuronal system, and 8 healthy controls. Unexpectedly, differences were not significant between glioma patients and healthy individuals/neuronal benign tumor patients, and between neuronal benign tumor patients and healthy controls (Figure 4A), suggesting that more clinical specimens should be examined. Although previous results support the high specificity and the great potentiality of serum DKK-1 as a biomarker for detection of myeloma/lung and esophageal carcinomas at an early stage and for monitoring of the relapse of the disease [17,19]. in patients with multiple glioma, serum concentrations of DKK-1 protein were close to the limit of detection by ELISA analysis due to the blood-brain barrier. It is also possible that certain substances with similar DKK-1 protein structure exist in sera and thus increase the background signal in ELISA experiments and reduce the expression differences between each group.

To evaluate the clinical usefulness of cerebral flucid DKK-1 level as a tumor detection biomarker, we also measured by ELISA the levels of DKK-1 protein in cerebral flucid samples from the same set of tumor patients and control individuals. The levels of cerebral fluid DKK-1 protein were significantly higher in glioma patients than in healthy donors or in neuronal benign tumor patients (P < 0.05); the difference between healthy individuals and neuronal benign tumor patients was not significant (Figure 4B), suggesting that the DKK-1 molecule secreted and stably expressed in cerebral fluids can also be applicable to detect presence of glioblastoma and to develop novel prognostic treatments.

## Discussion

Human DKK-1 is a member of the DKK gene family and maps to chromosome 10q11.2 [20]. DKK-1 is expressed in a timely and spatially controlled manner during development. It was first isolated in *Xenopus*, where it is expressed in the Spemann organizer as a head inducer [21], and its important role in normal head development in mice has also been identified [22]. Other members of the family are DKK-2, DKK-3, and DKK-4, which all contain two cysteine-rich domains that are highly conserved among different family members [18]. Although DKK-1 functions as an inhibitor of the Wnt signaling pathway [21], DKK-2 activates Wnt signaling in *Xenopus *embryos [23].

DKK-1 has multiple biological roles in a variety of cancers. The forced expression of DKK-1 in the small intestine inhibits cell proliferation and the generation of secretory lineages [24,25]. Furthermore, DKK-1 seems to induce the proliferation of human adult bone marrow stem cells [26] and contribute to the control of osteoporosis, as mutations in LRP5 that impede binding of DKK-1 are responsible for high bone density [27]. DKK-1 also inhibits osteoblastic differentiation and high circulating levels of DKK-1 in patients with multiple myeloma are associated with osteolytic lesions [28]. Gene expression profile analysis of lung and esophageal carcinomas revealed that DKK-1 was highly transactivated in the great majority of lung cancers and esophageal squamous cell carcinomas [17]. Overexpression of DKK-1 has also been detected in human hepatoblastomas and Wilms' tumors [29]. In contrast, expression of the DKK-1 gene, a downstream target of β-catenin/TCF, decreases in human colon tumors, indicating its tumor suppressing role in this neoplasia [1]. Recent evidence also suggests that DKK-1 is a functional suppressor of HeLa cell transformation [15].

Human DKK-1 was reported to be responsive to p53 [30], although it has been shown to be induced by DNA damage and to sensitize to apoptosis in a p53-independent manner [31]. Recently, glucocorticoids have been reported to enhance DKK-1 expression in human osteoblasts [32]. However, little is known about the control mechanism of DKK-1 expression in human gliomas.

Medulloblastoma is a heterogeneous pediatric brain tumor, and DKK-1 expression in primary medulloblastoma cells and patient samples by RT-PCR was found to be significantly down-regulated relative to normal cerebellum [33]. Transfection of a DKK-1 gene construct into D283 cell lines suppressed medulloblastoma tumor growth in colony focus assays by 60% (P < 0.001), and adenoviral vector-mediated expression of DKK-1 in medulloblastoma cells increased apoptosis fourfold (P < 0.001) [33]. In the present study, we observed that DKK-1 transcript and protein widely express in glioma cell lines and pathologic tumor tissues with increased levels but not in medulloblastoma cell line D341, indicating different expression pattern of DKK-1 in intracranial neuroepithelial carcinomas.

Although secreted Wnt antagonists have been found to be down-regulated or silenced in certain carcinomas [34-38], DKK-1 expression is restored in glioma cells. Our data suggest the possible roles of DKK-1- in carcinogenesis of gliomas. It remains unclear if the increased DKK-1 expression is in response to Wnt activation in gliomas or independent effect. Further detailed experiments will shed light on this interesting point.

## Conclusion

In this paper we report that the role of DKK-1, an inhibitor of the Wnt pathway, in gliomas. We demonstrate that DKK-1 is expressed by malignant glioma cells but not by other tumor cell lines investigated using RT-PCR and ELISA. Our findings are confirmed by immunohistochemical stainings of DKK-1 in glioma and normal human brain tissue. Elevated DKK-1 levels are also found in cerebrospinal fluid of glioma patients. Thus, we conclude that DKK-1 may have an important role in glioma tumorigenesis.

## Competing interests

The authors declare that they have no competing interests.

## Authors' contributions

FL carried out the molecular genetic studies, participated in the ELISA assay, and drafted the manuscript. QW carried out the immunoassays. QX participated in design of the study and performed the statistical analysis. YZ conceived of the study, and participated in its design and coordination and helped to draft the manuscript. All authors read and approved the final manuscript.
